# Structure of the *Lysinibacillus sphaericus* Tpp49Aa1 pesticidal protein elucidated from natural crystals using MHz-SFX

**DOI:** 10.1073/pnas.2203241120

**Published:** 2023-11-28

**Authors:** Lainey J. Williamson, Marina Galchenkova, Hannah L. Best, Richard J. Bean, Anna Munke, Salah Awel, Gisel Pena, Juraj Knoska, Robin Schubert, Katerina Dörner, Hyun-Woo Park, Dennis K. Bideshi, Alessandra Henkel, Viviane Kremling, Bjarne Klopprogge, Emyr Lloyd-Evans, Mark T. Young, Joana Valerio, Marco Kloos, Marcin Sikorski, Grant Mills, Johan Bielecki, Henry Kirkwood, Chan Kim, Raphael de Wijn, Kristina Lorenzen, Paul Lourdu Xavier, Aida Rahmani Mashhour, Luca Gelisio, Oleksandr Yefanov, Adrian P. Mancuso, Brian A. Federici, Henry N. Chapman, Neil Crickmore, Pierre J. Rizkallah, Colin Berry, Dominik Oberthür

**Affiliations:** ^a^School of Biosciences, Cardiff University, Cardiff CF10 3AX, United Kingdom; ^b^Center for Free Electron Laser Science, Deutsches Elektronen-Synchrotron, 22607 Hamburg, Germany; ^c^European XFEL GmbH, 22869 Schenefeld, Germany; ^d^Department of Biological Sciences, California Baptist University, Riverside, CA 92504; ^e^Max-Planck Institute for the Structure and Dynamics of Matter, 22761 Hamburg, Germany; ^f^Department of Chemistry and Physics, La Trobe Institute for Molecular Science, La Trobe University, Melbourne, VIC 3086, Australia; ^g^Department of Entomology and Institute for Integrative Genome Biology, University of California, Riverside, CA 92521; ^h^Centre for Ultrafast Imaging, Universität Hamburg, 22761 Hamburg, Germany; ^i^Department of Physics, Universität Hamburg, 22761 Hamburg, Germany; ^j^School of Life Sciences, University of Sussex, Falmer, Brighton BN1 9QG, United Kingdom; ^k^School of Medicine, Cardiff University, Cardiff CF14 4XN, United Kingdom

**Keywords:** XFEL, SFX, *Lysinibacillus sphaericus*, pesticidal protein, Tpp49Aa1

## Abstract

The *Lysinibacillus sphaericus* proteins Tpp49Aa1 and Cry48Aa1 can together act as a toxin toward the mosquito *Culex quinquefasciatus* and have potential use in biocontrol. Given that proteins with sequence homology to the individual proteins can have activity alone against other insect species, the structure of Tpp49Aa1 was solved in order to understand this protein more fully and inform the design of improved biopesticides. Tpp49Aa1 is naturally expressed as a crystalline inclusion within the host bacterium, and MHz serial femtosecond crystallography using the novel nanofocus option at an X-ray free electron laser allowed rapid and high-quality data collection to determine the structure of Tpp49Aa1 at 1.62 Å resolution. This revealed the packing of Tpp49Aa1 within these natural nanocrystals as a homodimer with a large intermolecular interface. Complementary experiments conducted at varied pH also enabled investigation of the early structural events leading up to the dissolution of natural Tpp49Aa1 crystals—a crucial step in its mechanism of action. To better understand the cooperation between the two proteins, assays were performed on a range of different mosquito cell lines using both individual proteins and mixtures of the two. Finally, bioassays demonstrated Tpp49Aa1/Cry48Aa1 susceptibility of *Anopheles stephensi*, *Aedes albopictus,* and *Culex tarsalis* larvae—substantially increasing the potential use of this binary toxin in mosquito control.

## Significance

Pesticidal proteins from bacteria, such as *Bacillus thuringiensis* and *Lysinibacillus sphaericus,* are widely used as biocontrol agents against various mosquito vectors of human disease. Tpp49Aa1/Cry48Aa1 from *L. sphaericus* are required as a pair to exert toxicity and are able to overcome resistance to currently marketed bioinsecticides. We used an emerging technique to obtain a high-resolution structure of Tpp49Aa1 from natively produced crystals. Cellular models confirmed that both proteins are required to elicit cell death, demonstrating the potential utility of these cells as models for better understanding Tpp49/Cry48 mechanism of action. Bioassays identified activity of the protein pair against three previously unrecognized mosquito targets. These data will help inform the future design and use of biological mosquitocides.

Pesticidal proteins produced by *Bacillus thuringiensis* and *Lysinibacillus sphaericus* constitute the major active ingredients in bioinsecticides and transgenic crops (1). Highly pathogenic strains of *L. sphaericus* have been commercially applied in the field to control mosquito vectors of human disease for many years. *L. sphaericus* activity is due to the expression of Tpp1Aa2 and Tpp2Aa2 proteins, which together constitute a potent mosquitocidal toxin (2, 3). Despite successful use of these strains, cases of resistance to this toxin have been identified (4, 5). To address this, *L. sphaericus* isolates exhibiting toxicity against Tpp1Aa2/Tpp2Aa2-resistant mosquito larvae were characterized (6, 7), leading to the identification of a new toxin comprising the proteins Cry48Aa1 and Tpp49Aa1 (6–8). The Cry48Aa/Tpp49Aa pair is produced by a number of *L. sphaericus* strains and is composed of Cry48Aa, a 135-kDa protein belonging to the 3-domain family of Crystal (Cry) proteins, and Tpp49Aa, a 53 kDa protein belonging to the family of Toxin_10 pesticidal proteins (Tpp) (7, 9). Both proteins are deposited in the form of natural, parasporal crystals during sporulation of the host bacterium. Mosquito toxicity bioassays have revealed that both components in combination are required for activity against *Culex quinquefasciatus* larvae (7, 8). The Cry48Aa1/Tpp49Aa1 toxin pair did not show toxicity to any of the other insect species previously tested, including Coleoptera, *Anthonomus grandis*, Lepidoptera, *Anticarsia gemmatalis, Spodoptera frugiperda,* and *Plutella xylostella*, and Diptera, *Aedes aegypti* and *Anopheles gambiae* (8). When administered in purified protein form, the potency of the Cry48Aa1/Tpp49Aa1 pair against *C. quinquefasciatus* (7) is comparable to that of the currently utilized Tpp1Aa2/Tpp2Aa2 toxin pair (10). Hence, Cry48Aa1/Tpp49Aa1 could be applied in the development of new pesticidal agents aimed at overcoming Tpp1Aa2/Tpp2Aa2 resistance.

In common with many other bacteria-derived pesticidal proteins, the mode of action of the Cry48Aa1/Tpp49Aa1 toxin pair begins with ingestion of the crystalline inclusions, followed by solubilization and proteolytic cleavage of the protoxins in the alkaline environment of the gut (7). Following activation, both Cry48Aa1 and Tpp49Aa1 bind specifically and with high affinity to the *C. quinquefasciatus* brush border membrane (11). Several classes of Cry48Aa1/Tpp49Aa1 binding proteins have been identified, including maltases, aminopeptidases, alkaline phosphatases, and metalloproteases (12). In addition, an alpha-glucosidase (Glu71) from *C. quinquefasciatus* has been identified as a putative receptor of Cry48Aa1 (13). Following membrane interaction, subsequent cytopathological effects including cytoplasmic and mitochondrial vacuolation, endoplasmic reticulum breakdown, and microvillus disruption are seen (14), culminating in insect death.

The cooperation of the two-component system is not well understood, however, dot blot assays suggest that Cry48Aa1 and Tpp49Aa1 are able to form a complex (11). Moreover, competition assays have indicated that the N-terminal region of Tpp49Aa1 (residues Asn49–Ser148) is responsible for binding Cry48Aa1, while the C-terminal region (residues Ser349–Asn464) is involved in membrane interaction (11). Mutagenesis studies have indicated the functional importance of three out of the four Tpp49Aa1 cysteine residues, which are required for full larvicidal activity against *Culex* larvae, as well as Cry48Aa1 binding (15). Although homology modeling has been applied to produce structural models of both Cry48Aa1 and Tpp49Aa1 (8, 16), their structures had not been experimentally resolved.

The development of X-ray free electron lasers (XFELs) has given rise to a new approach in protein crystallography, known as serial femtosecond crystallography (SFX). SFX introduces a stream of crystals into an XFEL beam, where the delivery of intense X-ray pulses of several femtosecond duration enables diffraction data to be collected at high exposures in a serial fashion before structural information is lost due to radiation damage (17–19). As such, diffraction data are not limited by the small crystal size of natural crystals. In the field of pesticidal proteins, SFX using microfocused X-ray pulses has previously been applied to solve the structures of the Cyt1Aa, Cry3Aa and Cry11 toxins from *B. thuringiensis,* and the Tpp1Aa2/Tpp2Aa2 toxin from *L. sphaericus* (20–23).

Here, we employed SFX at the SPB/SFX instrument (24) of the European XFEL to elucidate the structure of Tpp49Aa1 from natural nanocrystals. Complementary experiments conducted at varied pH also enabled investigation of the early structural events leading up to the dissolution of natural Tpp49Aa1 crystals—a crucial step in its mechanism of action. To gain insight into the collaboration of Cry48Aa1 and Tpp49Aa1, cell assays were performed using both the individual proteins and mixtures of the two. Finally, we performed mosquito larvae bioassays to demonstrate an expanded target range for control by Cry48Aa1/Tpp49Aa1. This study is significant for understanding the mechanism of action of the Cry48Aa1/Tpp49Aa1 two-component system, which itself has substantial capability for overcoming insect resistance.

## 1. Results and Discussion

In this study, pesticidal proteins are referred to according to the recently revised nomenclature system produced by the Bacterial Pesticidal Protein Resource Center (https://bpprc.org/ and *SI Appendix*, Table S1)—(9).

### 1.1. Structure Description.

Tpp49Aa1 nanocrystals (*SI Appendix*, Fig. S1), produced from a recombinant *B. thuringiensis* strain, were used for diffraction data collection. Use of the novel nanofocus option of the SPB/SFX beamline enabled the beam focus to be matched to the size of the Tpp49Aa1 crystals. In addition, the ability of the European XFEL to deliver the highest available X-ray intensity per pulse, as well as the unique MHz-pulse structure, allowed for data collection at high repetition rate and determination of the Tpp49Aa1 structure from a high quality, high redundancy dataset. In total 2,610,333 detector images were collected, of which 2,307,611 could be indexed (an exceptionally high indexing rate of >88 %). We selected the 500,000 strongest patterns according to CrystFEL for downstream processing and refinement. From these 500,000 patterns, 680,109 crystals could be indexed (with—multi in CrystFEL) in space group P2_1_2_1_2_1_ (a ~ 79.65; b ~ 83.11; c ~ 156.91 Å; α = β = γ = 90). Phasing was performed by molecular replacement using *L. sphaericus* Tpp1Aa2/Tpp2Aa2 (PDB 5FOY and PDB 5G37) and *L. sphaericus* Tpp2Aa3 (PDB 3WA1) as starting models. Manual building and refinement from the ensembled diffraction data led to a model with R_work_/R_free_ of 0.178/0.197 at 1.62 Å resolution (*SI Appendix*, Table S2). The electron density map (*SI Appendix*, Fig. S2) showed continuous density for residues 49–464 of the protein sequence.

**Fig. 1.**
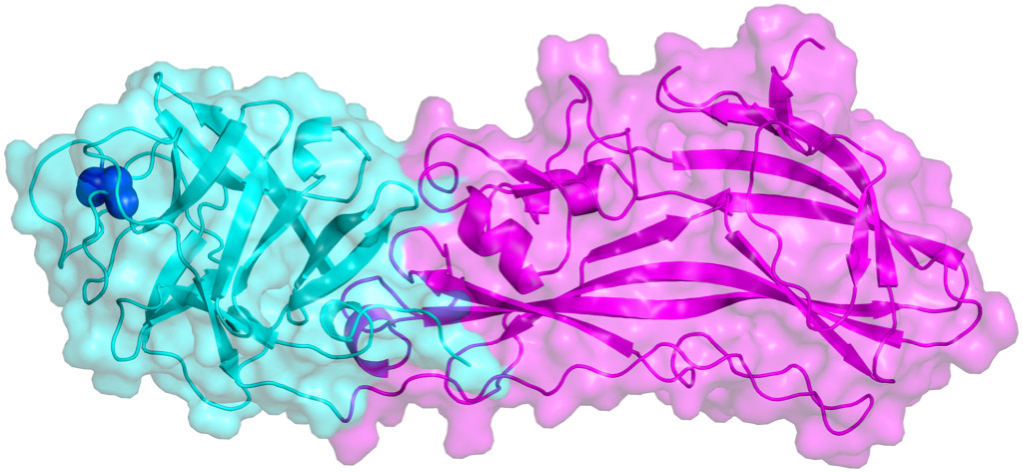
Crystal structure of Tpp49Aa1. Two distinct domains exist within the Tpp49Aa1 monomer. The N-terminal lectin-like head domain (cyan) consists of six β-hairpins that form a β-trefoil fold containing a disulfide bond Cys91–Cys183 (shown as dark blue spheres). The C-terminal pore-forming domain (magenta) comprises an aerolysin-like domain.

**Fig. 2.**
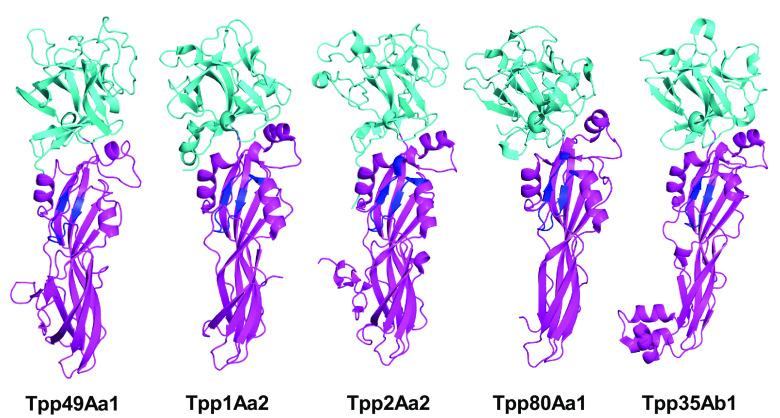
Comparative structures of β-sheet toxins. The structures of pesticidal proteins: Tpp49Aa1 (PDB 8BEY) from *L. sphaericus*, Tpp1Aa2 from *L. sphaericus* (PDB 5FOY—chain A), Tpp2Aa2 from *L. sphaericus* (PDB 5FOY—chain B), Tpp80Aa1 from *B. thuringiensis* (PDB 8BAD—chain A), and Tpp35Ab1 from *B. thuringiensis* (PDB 4JP0—chain A). Lectin-like domains are shown in cyan and pore-forming domains are shown in magenta. Regions including a short β-hairpin with predominantly amphipathic structure (shown in dark blue) were proposed as transmembrane domains in Tpp1 (residues 256–268) and Tpp2 (residues 302–317). A similar structure is found in the structure of Tpp35 (residues 249–259), Tpp80 (residues 259–272), and in our Tpp49 structure (residues 322–334).

Within each Tpp49Aa1 monomer (Fig. 1), two distinct domains exist: an N-terminal lectin-like head domain covering residues 49–214 and a C-terminal putative pore-forming domain (PFD) covering residues 215–464. Related structures were identified in the Protein Data Bank using the DALI server (25) with the best matches being other pesticidal proteins belonging to the Tpp family (PF05431), sharing both the common PFD and β-trefoil domains (Fig. 2): *L. sphaericus* Tpp2Aa2 (PDB 5FOY—chain B) (21), *L. sphaericus* Tpp2Aa3 (PDB 3WA1—chain A) (26), *L. sphaericus* Tpp1Aa2 (PDB 5FOY—chain A) (21), *B. thuringiensis* Tpp80Aa1 (PDB 8BAD—chain A) (27), and *B. thuringiensis* Tpp35Ab1 (PDB 4JP0—chain A) (16), with greatest structural similarity being between Tpp49Aa1 and Tpp2Aa2 (*SI Appendix*, Table S3).

The first 48 N-terminal residues are not seen in the structure, but are known to be present in Tpp49Aa1 crystals produced in the native host *L. sphaericus* (7). To ensure that this region was also present in our protein expressed in *B. thuringiensis*, N-terminal sequencing was performed. The resulting sequence MXNQ(E) corresponds to the authentic N-terminal sequence MENQI, confirming that the N terminus is intact and, therefore, apparently disordered in the crystals. This region is known to be proteolytically removed from the protein by *C. quinquefasciatus* gut extracts (8) and the structural flexibility that might disorder the sequence in the crystals may facilitate this activation of the protein. The N-terminal domain seen in the structure comprises a β-trefoil fold consisting of six two-stranded β-hairpins which form a β-barrel with a triangular cap, as well as one disulfide bond, Cys91-Cys183. The β-hairpins in this, and other β-trefoil folds, are arranged into three subdomains commonly designated α, β, and γ, giving rise to a pseudo-three-fold axis. β-trefoil folds are highly conserved in the lectin family, a class of carbohydrate-binding proteins (28). Several studies have indicated a role for carbohydrate moieties in eliciting pesticidal action of Cry proteins (29–33). Hence, it is possible that carbohydrate binding may also be important for pesticidal activity of the Cry48Aa1/Tpp49Aa1 toxin pair. In Tpp2Aa2 of the Tpp1Aa2/Tpp2Aa2 complex, the Cys67-Cys161 disulfide bond, which is the equivalent of the Cys91-Cys183 disulfide bond identified in the Tpp49Aa1 structure (*SI Appendix*, Fig. S3), has been proposed to obstruct the putative α sugar binding module (21). Elimination of the Cys residues in either Tpp2Aa3 (34) or in Tpp49Aa1 (15), eliminates toxicity.

The C-terminal domain of Tpp49Aa1 comprises a β sheet-rich topology characteristic of the aerolysin family of pore-forming toxins (35, 36). Indeed, Lacomel et al. speculated that the Tpp family may represent a subclass of the larger aerolysin, ETX/MTX-2 superfamily of β-pore forming proteins (37). Aerolysin, which is secreted as the inactive proaerolysin homodimer, is first activated by proteolytic cleavage of the C-terminal propeptide, allowing dissociation to a monomer (35, 38). The N-terminal receptor binding domains interact with N-glycosylated GPI-anchored receptors (39). Oligomerization of seven monomers, via interaction of the PFDs, leads to formation of a prepore structure constituting an amphipathic β-barrel, which inserts into the membrane, leading to cell death by osmotic lysis (38). The structural homology seen between Tpp49Aa1 and the aerolysin family may suggest that Tpp49Aa1 is able to form pores by a similar mechanism. In an analysis of the Tpp proteins and the wider aerolysin family (37), a region including a short β-hairpin with predominantly amphipathic structure, tucked under a loop within the PFD, was proposed as the transmembrane domain in Tpp1 (residues 256–268) and Tpp2 (residues 302-317) (Fig. 2). A similar structure is found in Tpp35Ab1 (residues 249–259), Tpp80Aa1 (residues 259–272), and in our Tpp49Aa1 (residues 322–334) (Fig. 2). We speculate that, as in an aerolysin-like mechanism, this region may unfold to form the β-barrel pore in the target cell membrane. However, it also cannot be ruled out that pore formation by Tpp49Aa1 may occur via unique mechanisms and the necessity for Cry48Aa1 in the action of Tpp49Aa1 raises further questions regarding the formation of pores by this protein pair.

**Fig. 3.**
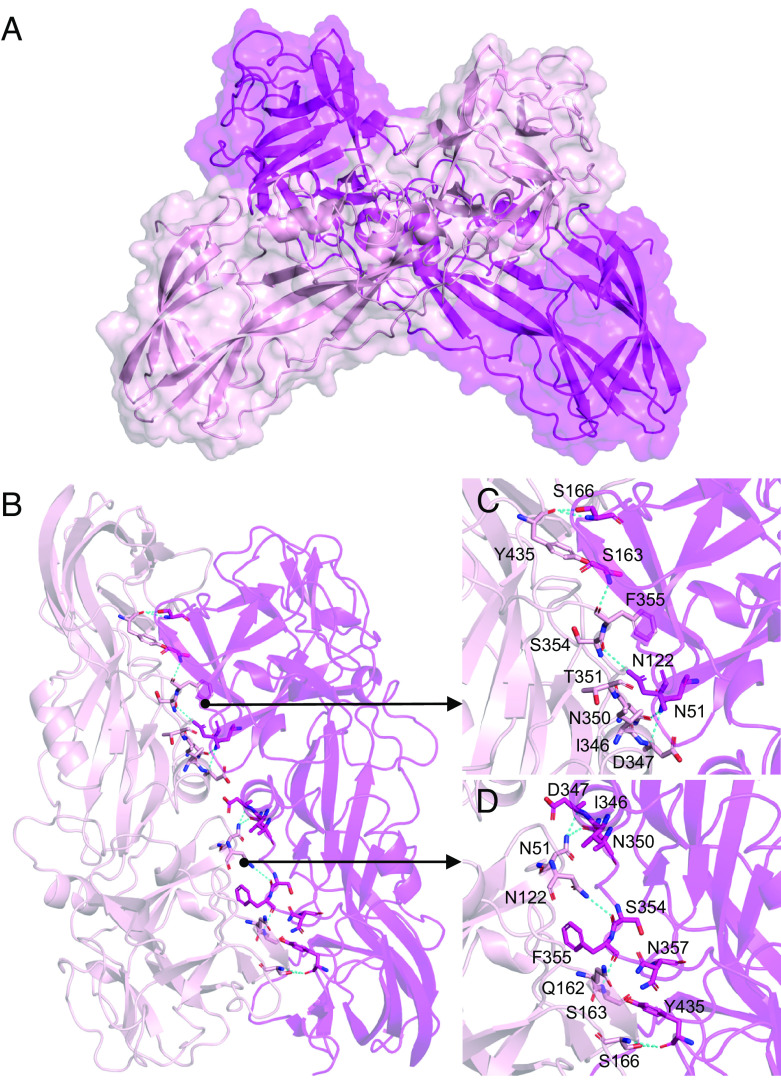
Structure of Tpp49Aa1 homodimer. (*A*) Tpp49Aa1 homodimer (monomer A—magenta, monomer B—light pink) forming an X structure with a large intermolecular interface. (*B*) Tpp49Aa1 dimer interface. Residues involved in hydrogen bonds identified by PDBePISA are shown as sticks (carbon—magenta/light pink, oxygen—red, nitrogen—blue). Polar interactions within a 3.6 Å cutoff are highlighted by cyan dashes. (*C* and *D*) Detailed view of interfacial residues involved in hydrogen bonding.

The final model of Tpp49Aa1 revealed the presence of a homodimer forming an “X” structure with a large intermolecular interface (Fig. 3), similar to that described for natural heterodimeric crystals of Tpp1Aa2/Tpp2Aa2 (*SI Appendix*, Fig. S3) (21). Superposition of the Tpp49Aa1 monomers shows the two copies to be almost identical, with an all-atom RMSD of 0.681 Å (*SI Appendix*, Fig. S4). The interface between the two monomers, which involves 41 residues from monomer A and 42 residues from monomer B, forming 16 hydrogen bonds (*SI Appendix*, Table S4 and Fig. 3), exhibits an area of 1329.1 Å^2^ and the predicted binding energy is −11.1 kcal mol^−1^ (*SI Appendix*, Table S4). In the Tpp1Aa2/Tpp2Aa2 heterodimer (*SI Appendix*, Fig. S3), the interface between the monomers involves 49 residues from Tpp1Aa2 and 63 residues from Tpp2Aa2, making 19 hydrogen bonds and 2 salt bridges. Interface analysis by PDBePISA estimates the area at 1833.1 Å^2^ and the binding energy at −22.5 kcal mol^−1^, indicating a more stable complex for Tpp1Aa2/Tpp2Aa2 heterodimers than for Tpp49Aa1 homodimers. Indeed, while interaction of the solubilized and activated forms of Tpp1Aa2/Tpp2Aa2 is required for full toxicity in the mosquito larvae, interaction of Tpp49Aa1 may only occur in the crystal to support packing and stability. These different interactions highlight an interesting feature of the Tpp family, in which some members (Tpp36, Tpp78, and Tpp80) can exert pesticidal activity alone, while others show co-dependency: Tpp1/Tpp2 requires interaction of two distinct members of the Tpp group, Tpp35 requires interaction with Gpp34, and Tpp49Aa1 requires interaction with Cry48Aa1, a member of a distinct (Cry) structural family.

To investigate whether the Tpp49Aa1 dimeric form is maintained in solution, solubilized Tpp49Aa1 was assessed using SEC (*SI Appendix*, *Supplementary Methods* 1). Tpp49Aa1 is present as two major bands that run at around 49 kDa and 55 kDa on an SDS-PAGE gel (*SI Appendix*, Fig. S5*A*). Solubilized Tpp49Aa1 crystals have previously been shown to produce similar size bands (7), and these may be products that are produced during analysis by bacterial proteinases present in the crystal preparation. SEC showed a main peak at 51 kDa, but with a shoulder peak before at 121 kDa indicating that it is predominantly a monomer but that some dimers may persist (*SI Appendix*, Fig. S5*B*). In further analysis, static light scattering and refractive index measurements were used to analyze the molecular weight of the protein-containing fractions (*SI Appendix*, *Supplementary Methods* 2). Calibrated to BSA, this indicated that Tpp49Aa1 is present in solution at a single MW corresponding to 52.1 kDa, approximately the weight of the monomer (*SI Appendix*, Fig. S5*C*). Both methods discussed above indicate that Tpp49 is predominantly present as a monomer in solution at pH 8.5. Within the natural crystals, the N-terminal region of Tpp49Aa1 (residues Asn49–Ser148) that has been suggested to interact with Cry48Aa1 (11), is partially buried within the large intermolecular interface (*SI Appendix*, Fig. S6). Dissociation of the Tpp49Aa1 dimer would, therefore, expose this region for interaction with Cry48Aa1.

### 1.2. Alkaline-Triggered Release of Tpp49Aa1.

An important feature that Tpp49Aa1 shares with a range of other pesticidal proteins in the taxonomic class Bacilli is its propensity to form natural crystals (when expressed at high level in its source organism, *L. sphaericus,* and in this study, in recombinant form in *B. thuringiensis*) and be solubilized in the pH of the insect gut. Tetreau et al., have recently reviewed factors within proteins that may contribute to the ability of bacteria to sequester them into natural crystals (40). Natural crystals of Cry3Aa (PDB 4QX0) (20) were found to have a high solvent content: 60.4% by Matthews’ analysis compared to a mean of ~47% in a previous analysis of in vitro grown crystals of proteins in the PDB (41). Tpp49Aa1 has an estimated 49.6% solvent content—closer to the mean of in vitro grown crystals—but, like natural crystals of Cry3Aa, the Tpp49Aa1 crystals are permeated by wide solvent accessible channels (*SI Appendix*, Fig. S7). We can speculate that the solvent channels within the crystals may facilitate dissolution in the appropriate environment.

The ability of natural crystals to remain stable in the open environment but to be dissolved by insect gut pH changes is thought to rely on a range of factors, including intermolecular salt bridges that may be pH labile, intermolecular hydrogen bonds that may be deprotonated upon pH elevation leading to electrostatic repulsion, and other gut solutes that may affect solubilization. PDBePISA analysis shows that the packing of dimers into crystals introduces eight possible interfaces between neighboring dimers (*SI Appendix*, Table S4). A network of hydrogen bonds and salt bridges is also observed. Given that Tpp49Aa1 crystals are readily solubilized in the alkaline environment of the mosquito larval gut, we next sought to investigate the effect of extreme pH on Tpp49Aa1 crystals. To do so, pH 11 buffer was injected into the vacuum chamber of the SPB/SFX beamline, allowing mixing with the natural Tpp49Aa1 crystals prior to data collection. In total, 707,992 diffraction patterns were collected from which 426,506 could be indexed in space group P2_1_2_1_2_1_, (a ~ 80.12; b ~ 83.22; c ~ 156.49 Å; α = β = γ = 90, highlighting an increase in unit-cell dimensions in comparison to the pH 7 dataset) (*SI Appendix*, Table S2). Phasing was performed by molecular replacement using the native (pH 7) Tpp49Aa1 structure as the starting model. Manual building and refinement from the ensembled diffraction data led to a model with R_work_/R_free_ of 0.177/0.199 at 1.75 Å resolution (*SI Appendix*, Table S2). Again, the electron density map (*SI Appendix*, Fig. S2) showed continuous density for residues 49–464 of the protein sequence and, as in the map obtained for the pH 7 dataset, the first 48 N-terminal residues were not observed.

The pH 7 and pH 11 structures align closely, displaying an all-atom RMSD of 0.487 Å. As in the Tpp1Aa2/Tpp2Aa2 structure (21), an increase in pH from 7 to 11 is estimated to alter the net charge of Tpp49Aa1 from −6.518 to −47.822 e, suggesting that alkalinity will destabilize the crystal by negative electrostatic repulsion. Within the Tpp49Aa1 monomer, the largest conformational changes occurred within the surface-exposed loops of the Tpp49Aa1 monomer, suggesting that these regions are affected by pH. Alkalinity also perturbed the packing of Tpp49Aa1 dimers into crystals, identified by analysis of crystal contacts and interfacial interactions using PDBePISA (*SI Appendix*, Tables S4–S6). Overall, the binding energy of all eight crystal contacts (excluding the dimer interface) increases by 1.8 kcal mol^−1^, indicating a weaker affinity. Despite this, the binding energy of the dimer interface decreases (pH 7 = −11.1, pH 11 = −11.9 kcal mol^−1^), indicating an increase in affinity. This may suggest that, during dissolution, contacts between individual dimers dissociate prior to the dimers themselves. Subsequent release of the Tpp49Aa1 monomers will expose the regions required for activation and interaction with the target membrane and with its partner protein, Cry48Aa1.

Alkalinity also induces changes in 18 interfacial interactions at both the dimer interface and crystal contacts (*SI Appendix*, Table S7). The most notable changes include loss of hydrogen bonds Asn51[ND2]-Asn350[OD1] and Asn51[ND2]-Asp347[O] of both monomers at the dimer interface. Given the proximity of these interactions to the cleavage site (Phe48) of the N-terminal propeptide, we hypothesize that loss of these hydrogen bonds would increase accessibility of the propeptide to proteases. In addition, elevated pH leads to loss of a salt bridge, Asp429[OD1]-Arg267[NH1], at a crystal contact outside of the dimer interface, supporting the idea that crystal stability is thought to rely, in part, on intermolecular salt bridges. Other changes in the interactions have been listed in *SI Appendix*, Table S7.

Diffraction data were also collected following mixing with pH 3 buffer. In total, 466,741 diffraction patterns were collected from which 279,362 could be indexed in space group P2_1_2_1_2_1_, (a ~ 79.89; b ~ 82.84; c ~ 157.88 Å; α = β = γ = 90, highlighting an increase in unit-cell dimensions in comparison to the pH 7 dataset) (*SI Appendix*, Table S2). Manual building and refinement from the ensembled diffraction data led to a model with R_work_/R_free_ of 0.189/0.213 at 1.78 Å (*SI Appendix*, Table S2). Similar changes in the structure were seen following a decrease in pH from 7 to 3 (*SI Appendix*, Tables S5, S8), although this is not physiologically relevant to pH values found in the target insect guts. Briefly, mixing at pH 3 results in a larger increase in overall binding energy at the eight crystal contacts (5.0 kcal mol^−1^) in comparison to mixing at pH 11 (1.8 kcal mol^−1^), suggesting that crystals may dissolve more readily at low pH. These studies shed light on the early events leading up to the dissolution of natural Tpp49Aa1 crystals.

### 1.3. Cellular Models of Cry48Aa1 and Tpp49Aa1 Action.

**Fig. 4.**
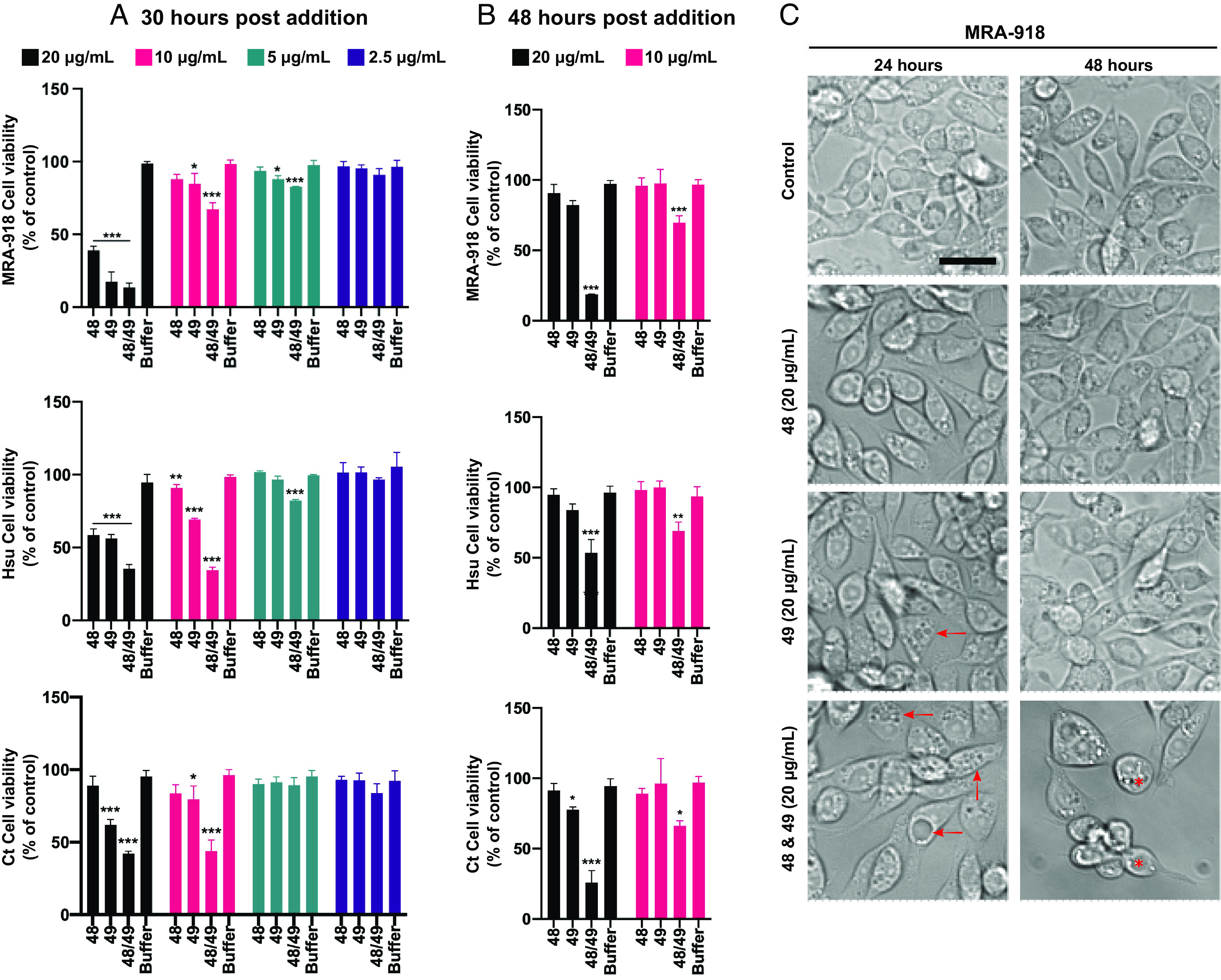
Cry48Aa1 and Tpp49Aa1 reduce cell viability in *C. quinquefasciatus* and *C. tarsalis* cell lines. To assess the use of various insect cell lines for modeling Cry48Aa1 and Tpp49Aa1 toxicity, resazurin was used to quantify the effect of these proteins on cell viability. *C. quinquefasciatus* (MRA-918, Hsu) and *C. tarsalis* (Ct) cells were treated with a range of concentrations of either Cry48Aa1 (“48”), Tpp49Aa1 (“49”), or equimolar amounts in combination (48/49). Resazurin was added to the cells 24 h (*A*) and 48 h (*B*) after the pesticidal proteins. (*A*) Twenty-four hours post exposure, reduced cell viability (% of control) was observed in all cell lines, with either 48/49 in combination or alone. (*B*) Twenty-four hours post 48/49 exposure, reduced cell viability is still seen with both pesticidal proteins (alone and in combination), however, the decreased viability between the single protein treatments is reduced compared to the combination, indicating recovery. All data are presented as the mean ± SD and all statistical analysis was performed using a one-way ANOVA, *n* = 3 (***P < 0.001, **P < 0.01, and *P < 0.05). (*C*) Brightfield images of MRA-918 cells at 24 h, post pesticidal protein addition, show a slightly rounded cellular morphology with either 48 or 49, alongside the presence of some small vacuoles in 49 alone (red arrows). Larger vacuoles are apparent in 48/49 combination (red arrows). At 48 h post 48/49 addition, cells have rounded and detached from the plate (red asterisk). Representative scale bar = 10 μm.

The cellular toxicity of Cry48Aa1 and Tpp49Aa1 was investigated in two cell lines derived from the known target species *C. quinquefasciatus* (MRA-918 and Hsu), and a cell line from another untested mosquito—*Culex tarsalis* (Ct). To investigate cytotoxicity, resazurin was used as an indicator of metabolic activity and cellular viability (42). Cry48Aa1/Tpp49Aa1 treatment reduced cellular metabolism in all three cell lines, confirming a functional effect of the proteins generated in this study (Fig. 4).

In cells from the known target species, *C. quinquefasciatus*, exposure to a 20 μg/mL total concentration of either the individual proteins, or a 1:1 w/w combination, resulted in significantly reduced metabolic activity after 24 h in both cell lines (Fig. 4*A*). By 48 h, however, cells exposed to the individual proteins had recovered, whereas loss of activity was still observed for the 1:1 mixture (Fig. 4*B*). A similar pattern was observed for the *C. tarsalis*-derived cell line (Ct) suggesting that this insect might also be susceptible to the toxin. At the lower concentration of 10 μg/mL, Cry48Aa1 alone had little or no effect on the three cell lines, whereas Tpp49Aa1 alone had a small effect at 24 h, which was then again not apparent at 48 h. As with the higher 20 μg/mL concentration the 10 μg/mL combination had significant effects at both 24 h and 48 h. Lower tested concentrations had minimal effect on the cells. No effects on metabolic activity or cellular morphology were apparent following addition of the proteins to Sf9 cells (*SI Appendix*, Fig. S8), consistent with the fact that Cry48Aa1/Tpp49Aa1 is reported nontoxic to the source insect—*S. frugiperda*.

Light microscopy of the MRA-918 cell line showed that the Cry48Aa1/Tpp49Aa1 combination—and to a lesser degree Tpp49Aa1 alone—can induce vacuolization at 24 h post exposure (Fig. 4*C*). This phenotype appears to recover in the Tpp49Aa1 only treated cells at 48 h post exposure, whereas in the Cry48Aa1/Tpp49Aa1 treatment we observed a rounded morphology, retraction of cellular processes and significant detachment from the plate, indicative of cell death. In agreement with our viability assays, and previous research (7, 8), this indicates both components are required for cell death. Cytoplasmic and mitochondrial vacuolization has been reported in previous studies administering Cry48Aa1/Tpp49Aa1, in combination, to midgut epithelial cells isolated from *C. quinquefasciatus* larvae (14). Similar phenotypes are also reported with Tpp49Aa-related Toxin_10 family members Tpp1Aa1/Tpp2Aa2 in both larvae (43, 44) and in cells expressing the relevant Cpm1 receptor (45).

We had previously demonstrated that neither individual protein showed activity against *C. quinquefasciatus* mosquito larvae (7) whereas in the cell models utilized here some, albeit transient, activity was seen at high concentrations. Cell lines have previously been shown to represent good model systems for the analysis of pesticidal proteins although various limitations have been described (46). In particular, it should be noted that Hsu is an ovary derived cell line and MRA-918 are from neonate larvae. We know the site of action for these toxins is the mosquito midgut, and the expression level of the relevant receptor(s) in the cell lines is yet to be determined. Nonetheless, the observation that mosquito-derived lines were susceptible while the *Spodoptera* one was not, correlates with known target specificity (8). In contrast to the irreversible cytocidal effects of both proteins combined, the transient changes seen with individual components may indicate a different mode of action or that the presence of the combination may enhance binding or overcome cellular defense and repair mechanisms. A number of different models have been proposed to explain synergistic interactions between two different toxins including heteroligomerization (47) and surrogate receptors (48). In the latter model a non, or weakly active, toxin can bind to the target cell and act as a receptor for a different toxin and thus increase the effect of that toxin. A possible model then for the joint action of Cry48Aa1 and Tpp49Aa is that one can act as a surrogate receptor for the other. Such a model would be consistent with the observations that both proteins are required for good activity, that both have been shown to bind to the midgut of a susceptible host and that preliminary evidence for a physical interaction has been provided in the form of dot blots (11).

### 1.4. Insect Bioassays.

We had previously found that the Cry48Aa1/Tpp49Aa1 pair only showed toxicity toward one of the eight tested insect species (8). The observation above that it showed activity against a cell line from *C. tarsalis* prompted us to test the activity of a 1:1 (w/w) mixture against the larvae of further mosquito species, namely: *Aedes albopictus, Anopheles stephensi,* and *C. tarsalis*. Cry48Aa1/Tpp49Aa1 demonstrated toxicity against all three species, with LC_50_ values of 111 ng/mL, 173 ng/mL, and 91 ng/mL at 48 h, respectively (Table 1). We also confirmed that the toxin pair still had activity against *C. quinquefasciatus* but not against *Ae. aegypti* or *An. gambiae*. After 24 h exposure to a high dose of the Cry48Aa1/Tpp49Aa1 pair, we observed mortality in the *C. quinquefasciatus* larvae. A high dose of Cry48Aa1/Tpp49Aa1 caused no mortality in *Ae. aegypti* or *An. gambiae* (nor did a high dose of the individual toxin components). The three new mosquito species are significant vectors of human disease, with *Ae. albopictus* reported as a primary vector for emerging arboviruses (49)—such as dengue virus—and *C. tarsalis* suggested as the most important vector of human West Nile virus in the US upper midwest region (50). Furthermore, *An. stephensi* was recently reported as a new malaria vector in Africa. *An. stephensi* is one of the few anopheline species found in central urban locations, putting 126 million Africans at risk, and prompting the World Health Organization to issue a vector alert and call for targeted control and prioritized surveillance (51).

**Table 1.** Potency of a 1:1 mixture of Cry48Aa1/Tpp49Aa1 to fourth instar mosquito larvae

Mosquito species	Exposure (h)	LC_50_ (ng/mL) (95% fiducial limits)	LC_50_ (ng/mL) (95% fiducial limits)
*Ae. albopictus*	24	607 (332–1,110)	4,800 (2,630–8,800)
	48	111 (68–183)	1,360 (823–2,230)
*An. stephensi*	24	818 (450–1,490)	3,830 (2,100–6,960)
	48	173 (118–253)	712 (450–965)
*C. tarsalis*	24	1,890 (578–6,160)	301,000 (92,100–982,000)
	48	91 (61–134)	436 (295–646)

## 2. Conclusions

We have used megahertz SFX at the European XFEL to determine the Tpp49Aa1 structure to a final resolution of 1.62 Å, thus emphasizing the utility of this approach in generating high-resolution structures from in vivo grown natural crystals. The protein was found to exist in dimeric form within the crystal and complementary experiments conducted at varied pH indicated that during solubilization of the crystals, the dimers first separate from themselves before they themselves are separated to release monomers. Assays involving susceptible cell lines indicated that while at high concentrations the individual proteins show some morphological changes, a high level of toxicity is only seen when both components are present. Two-component toxins are not uncommon, with classical AB toxins consisting of an active catalytic unit and a binding unit, the role of which is to translocate the active part into the cell (52). Cry48Aa1 and Tpp49Aa1 do not fit this model since neither is catalytic and both individually appear to represent pore-forming toxins. Two component pore-forming toxins do exist, such as YaxAB (53) where hetero-oligomers of the two proteins make up the pore. While this is possible in our system, it seems unlikely given that Cry48Aa1 resembles an α-pore-forming toxin and Tpp49Aa1 a β-pore-forming toxin. Another possibility is that one of the proteins acts as a surrogate receptor for the other, as has previously been described for other mosquitocidal toxins (54, 55). Our previous observation that Tpp49Aa1 toxicity is optimum in the presence of equimolar concentrations of Cry48Aa1 (8) is consistent with such a model. If such a system has evolved naturally to target new insect hosts, then the structure described here may contribute to future design of new synergistic partnerships to develop novel insecticidal pairs.

## 3. Materials and Methods

### 3.1. Purification of Tpp49Aa1 Protein Crystals.

The *B. thuringiensis* recombinant strain 4Q7::pHTP49, encoding the *tpp49Aa1* gene (accession number AJ841948) (7), was grown in 400 mL Embrapa medium (56) containing 5 μg/mL erythromycin at 30 °C with shaking (200 rpm) until sporulation reached >90%, as judged by phase contrast microscopy. Sporulated cultures were harvested and the natural crystal proteins were isolated using stepped sucrose gradients as previously described (7). Crystal protein was run on SDS-PAGE and blotted onto PVDF membrane for N-terminal sequencing by Alta Bioscience Ltd (Redditch). For use in bioassays, Cry48Aa1 crystals were prepared from the recombinant Bt strain 4Q7::pSTABP135 (7) using the method described above.

### 3.2. Bioassays.

Bioassays were carried out against a range of insects (*C. quinquefasciatus*, *Ae. aegypti* and *An. gambiae, Ae. albopictus, An. stephensi, and C. tarsalis*) and the cell lines *C. quinquefasciatus* (Hsu, and MRA-918) *C. tarsalis* (Ct), and *S. frugiperda* (Sf9) (57–59). It should be noted that the MRA-918 line (also known as 4A3A) was originally reported as an *An. gambiae* line but the cells have been independently verified by a number of laboratories, including our own (amplifying and sequencing mitochondrial large ribosomal subunit, mitochondrial cytochrome C oxidase, and maltase genes), to be *C. quinquefasciatus.*

To confirm previous findings, a high dose of Tpp49Aa1/Cry48Aa1 was given either together or separately to *C. quinquefasciatus*, *Ae. aegypti* and *An. gambiae.* Toxins were added to 1 mL of water containing five third instar larvae that were checked for mortality 24 h later. To investigate new potential targets for Tpp49Aa1/Cry48Aa1, *Ae. albopictus*, *An. stephensi,* and *C. tarsalis* larvae were bioassayed using 350 mL cups, each containing 100 mL of distilled water with 10 fourth-instar larvae. A range of concentrations of a 1:1 ratio w/w Cry48:Tpp49 were tested, one concentration per cup, with three replicates per concentration. Mortality was determined at 24 and 48 h. LC_50_ and LC_95_ values were determined using Finney’s probit analysis.

Insect cell lines were maintained at 27 °C in an appropriate growth medium which was changed every 4 d: Hsu, MRA-918, Ct (Schneider’s Insect Medium supplemented with 10% FBS) and Sf9 (Grace’s Insect Medium supplemented with 10% FBS). For cellular bioassays, cells were plated at 10,000 cells per well of a 96-well plate in 150 μL of medium and left to reach ~80% confluency. Tpp49Aa1/Cry48Aa1 proteins were solubilized in 50 mM Na_2_CO_3_ pH 10.5 + 0.05% β-mercaptoethanol. Solubilized toxin was treated with immobilized TPCK Trypsin (Thermo Scientific, 20230) overnight at 37 °C, followed by separation from the protein sample by centrifugation, and added either separately, or at a 1:1 molar ratio, in a range of concentrations (20, 10, 5, 2.5 μg/mL total protein) with the equivalent amount of solubilization buffer added into control wells (at no more than 5% of total well volume). As a measure of cell viability, resazurin (10 μg/mL) was added into the cell medium (10% v/v) at 24 h or 48 h post Cry48Aa1 and Tpp49Aa1 addition. Fluorescence was quantified using a Molecular Devices Spectramax Gemini EM plate reader (*λ*_ex_ = 445 nm: *λ*_em_ = 585 nm), 6 h post resazurin addition. Statistical analyses were performed using GraphPad Prism for Mac OS (Ver 8.2.0), using one-way ANOVAs followed by Dunnett’s multiple comparisons test to compare individual treatment groups back to the control. Data are presented as mean ± SD. For imaging, MRA-918 cells were seeded in 8-well Ibidi chamber slides (Thistle, IB-80826). Brightfield images were acquired with a Zeiss AX10 inverted microscope with AxioCam MRm camera and Axiovision 4.5.2 software (Zeiss, Cambridge).

### 3.3. Transmission Electron Microscopy.

Purified crystal batches were characterized using transmission electron microscopy (JEM 2100-Plus, JEOL) in the XBI lab of European XFEL (60). Holey carbon copper grids (Quantifoil R1.2/1.3) were glow discharged (GloQube Plus, Quorum Technologies) freshly before use. Crystal slurry (2 µL) was applied onto the grid and incubated for 30 s and blotted using filter paper (Whatman #1). Samples were negatively stained by placing the grid on a droplet containing 2% (w/v) uranyl acetate and blotted immediately. Grids were placed on a second uranyl acetate droplet and incubated for 20 s before blotting again and left to dry on filter paper. Samples were imaged with the TEM at 200 kV acceleration voltage using an Emsis Xarosa camera in imaging and selected area electron diffraction mode.

### 3.4. Structure Determination.

Crystals were washed with ddH_2_O and filtered through a cascade of nylon mesh filters (Sysmex CellTrics), ranging from 100 µm down to 5 µm mesh size. The crystal suspension was centrifuged at 200×*g* for 1 min and the supernatant—containing the Tpp49Aa1 nanocrystals—was subjected to another cascade of filtration and washing before transfer to the high-pressure sample reservoirs for injection into the XFEL beam. For pH studies, 0.1 M sodium citrate (pH 3.0) and 0.1 M sodium carbonate (pH 11.0) buffers were used. Buffers were transferred to the high-pressure sample reservoirs for injection and mixed with the Tpp49Aa1 crystals 1 m upstream of the XFEL beam, approximately 1 min before probing with X-rays. Megahertz serial femtosecond crystallography (61, 62) diffraction data were collected at the SPB/SFX (24), instrument of the European XFEL facility, Hamburg, Germany, using fast liquid-jet based injection (63) with 3D-printed (64) DFFN (65). With this set-up, 202 images per X-ray pulse train (with 10 trains/s repetition rate) were recorded with the AGIPD Detector at an intratrain pulse rate of 0.564 MHz. A photon energy of 9.3 keV with an average of 4 mJ/pulse was delivered to the instrument, focused to a spot size of about 300 nm diameter using the Nanoscale-focusing KB optics (66), providing about 6 × 10^12^ photons/µm^2^/pulse at the sample. The online crystal diffraction “hit-rate” was monitored using OnDA program (67) with raw data processing largely following the method described by Wiedorn et al. (61). Hit finding was performed using the program Cheetah (68) with careful optimization of the peak search parameters (--peaks=peakfinder8 --min-snr=5 --max-res=800 --threshold=500 --min-pix-count=1 --max-pix-count=50 --min-peaks=10 --local-bg-radius=3) and masking of bad pixels. Meaningful diffraction patterns were then indexed using CrystFEL (69, 70) version 0.10.1 (--int-radius=2,4,6, --multi) using the indexing method XGandalf (71). For the detector geometry optimization the program Geoptimizer was used (72). Merging and scaling of the integrated reflection intensities was performed using the Partialator program from the same CrystFEL package [--model=xsphere --min-res=3 --push-res=1.0 for pH 3 and pH 11, --model=unity --min-res=2.5 --push-res=inf for the native (pH 7) dataset]. The solvent content and number of molecules in the asymmetric unit was estimated using Matthews’ analysis, available through the MATTPROB web server (41). The phasing pipeline MRage in Phenix (73, 74) was used for initial phasing, using the sequence information and a component stoichiometry of two as input. MRage used models of *L. sphaericus* Tpp1Aa2/Tpp2Aa2 (PDB 5FOY and PDB 5G37) and *L. sphaericus* Tpp2Aa3 (BinB variant, PDB 3WA1) as templates for molecular replacement. The initial model was optimized using phenix.phase_and_build, followed by another round of Phaser and automatic model building using phenix.autobuild (75). The resulting model and maps were inspected manually using coot (76), followed by iterative refinement and model building cycles using phenix.refine (77) and coot respectively. In the initial molecular replacement, two noncrystallographic symmetry (NCS)-related domains A1 and A2 were switched (A1B2, A2B1), this error was corrected in coot and the residues linking A and B were built manually. This model was subjected to another cycle of phenix.autobuild, followed by iterative refinement and model building cycles using phenix.refine and coot respectively. Final refinement was carried out using Refmac5 (78) in the CCP4i2 package (79) keeping the R_free_-Flags generated in Phenix. The PDBePISA web server (80) was used to analyze the interfacial interactions of the Tpp49Aa1 crystals. PISA enabled calculation of the interface area (Å^2^) and Δ^i^G (kcal mol^1^), as well as the identification of interfacial hydrogen bonds and salt bridges.

## Supplementary Material

Appendix 01 (PDF)

## Data Availability

All study data are included in the article and/or *SI Appendix*. Crystallographic data are deposited with the Protein Databank under accessions 8BEY (81), 8BEX (82), 8BEZ (83). Raw data are available at https://zenodo.org/records/10148782 (84).
