# Supplementary material for: Structure of the Lysinibacillus sphaericus Tpp49Aa1 pesticidal protein elucidated from natural crystals using MHz-SFX
Source: Proc Natl Acad Sci U S A. 2023 Nov 28;120(49):e2203241120. doi: 10.1073/pnas.2203241120 (PMC10710082; doi:10.1073/pnas.2203241120)
Supplement: Supplementary file 1 — Appendix 01 (PDF) [file pnas.2203241120.sapp.pdf]

PNAS
www.pnas.org

Supplementary Information for

Structure of the *Lysinibacillus sphaericus* Tpp49Aa1 pesticidal protein elucidated from natural crystals using MHz-SFX

Authors
Lainey J. Williamson [1], Marina Galchenkova [2], Hannah L. Best [1], Richard J. Bean [3], Anna Munke [2], Salah Awel [2], Gisel Pena [2], Juraj Knoska [2], Robin Schubert [3], Katerina Döerner [3], Hyun-Woo Park[4], Dennis K. Bideshi [4], Alessandra Henkel [2], Viviane Kremling [2], Bjarne Klopprogge [2], Emyr Lloyd-Evans [1], Mark T. Young [1], Joana Valerio [3], Marco Kloos [3], Marcin Sikorski [3], Grant Mills [3], Johan Bieleki [3], Henry Kirkwood [3], Chan Kim [3], Raphael de Wijn [3], Kristina Lorenzen [3], P. Lourdu Xavier [2,5], Aida Rahmani [2], Luca Gelisio [2], Oleksandr Yefanov [2], Adrian P. Mancuso [3,6], Brian A. Federici[7], Henry N. Chapman [2,8,9], Neil Crickmore [10], Pierre J. Rizkallah [11], Colin Berry [1*]and Dominik Oberthür [2*]

Affiliations
[1] School of Biosciences, Cardiff University, UK; [2] Center for Free Electron Laser Science CFEL, Deutsches Elektronen-Synchrotron DESY, Notkestr. 85, 22607 Hamburg, Germany; [3] European XFEL GmbH, Schenefeld, Germany; [4] Department of Biological Sciences, California Baptist University, USA [5;] Max-Planck Institute for the Structure and Dynamics of Matter, 22761 Hamburg, Germany; [6] Department of Chemistry and Physics, La Trobe Institute for Molecular Science, La Trobe University, Melbourne, Victoria 3086, Australia; [7] Department of Entomology and Institute for Integrative Genome Biology, University of California, USA; Centre for Ultrafast Imaging, Universität Hamburg, Hamburg, Germany; [9] Department of Physics, Universität Hamburg, Hamburg, Germany; [10] School of Life Sciences, University of Sussex, Falmer, UK; [11] School of Medicine, Cardiff University, UK

Correspondence: berry@cardiff.ac.uk, dominik.oberthuer@cfel.de

This PDF file includes:

Figures S1 to S8
Tables S1 to S8
Supplementary methods 1
Supplementary methods 2

Table S1. Nomenclature system produced by the Bacterial Pesticidal Protein Resource Center (bpprc.org) and used in this work with older names for reference.

Current name assigned by BPPRC	Former name
Cry48Aa1	Cry48Aa1
Tpp49Aa1	Cry49Aa1
Tpp1Aa2	BinA2
Tpp2Aa2	BinB2
Tpp2Aa3	BinB3
Tpp35Ab1	Cry35Ab1
Tpp36	Cry36
Tpp78	Cry78
Tpp80Aa1	Cry80Aa1
Gpp34	Cry34
Cyt1Aa	Cyt1Aa
Cry3Aa	Cry3Aa
Cry11	Cry11

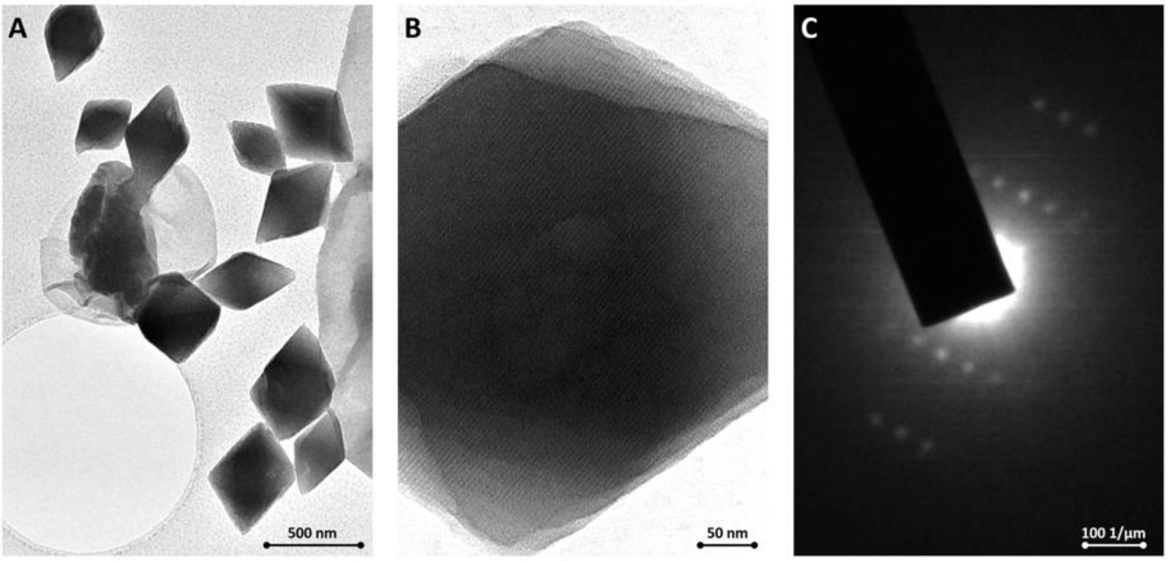

Figure S1. Transmission electron microscopy on negative stained Tpp49Aa1 nanocrystals. **(A)** Tpp49Aa1 nanocrystals and remaining parasporal bodies from the crystal purification process. **(B)** Visualization of crystal lattice of Tpp49Aa1 nanocrystals allows assessment of the quality of different purification batches. **(C)** Confirmation of crystal quality is obtained by selected area electron diffraction imaging (SAED).

Table S2. Data collection and refinement statistics for Tpp49Aa1.

**Data Collection**			
PDB ID	8BEX	8BEY	8BEZ
Beamline	SPB/SFX at European XFEL	SPB/SFX at European XFEL	SPB/SFX at European XFEL
X-ray Energy (keV)	9.3	9.3	9.3
Wavelength (Å)	1.33	1.33	1.33
**Crystal Data (figures in brackets refer to outer resolution shell)**			
pH	3	7	11
a,b,c (Å)	79.89, 82.84, 157.88	79.65, 83.11, 156.91	80.12, 83.22, 156.49
α,β,γ (°)	90.0, 90.0, 90.0	90.0, 90.0, 90.0	90.0, 90.0, 90.0
Space group	$P\ 2_1\ 2_1\ 2_1$	$P\ 2_1\ 2_1\ 2_1$	$P\ 2_1\ 2_1\ 2_1$
Resolution (Å)	1.78 – 36.01	1.62 - 24.98	1.75 – 36.12
Outer shell	1.78 – 1.80	1.62-1.64	1.75-1.77
R-split (%)	8.59 (231.27)	6.07 (299.67)	6.40 (177.89)
CC*	0.999 (0.492)	0.999 (0.484)	0.999 (0.632)
CC1/2	0.997 (0.138)	0.997 (0.133)	0.998 (0.249)
I / σ(I)	10.50 (0.81)	10.61 (0.36)	14.81 (0.75)
Completeness (%)	99.51 (92.76)	100 (100)	99.82 (97.34)
Multiplicity	183.70 (6.3)	1028.30 (186.7)	323.70 (10.6)
Total Measurements	18469814 (38681)	136507469 (1635083)	34281129 (71761)
Unique Reflections	100541 (6177)	132750 (8756)	105904 (6782)
Wilson B-factor($Å^2$)	27.96	21.79	23.82
**Refinement Statistics**			
Refined atoms	7,234	7,357	7,324
Protein atoms	6,867	6,877	6,854
Non-protein atoms	0	0	0
Water molecules	367	480	470
R-work reflections	95,333	130,631	100,468
R-free reflections	5,033	6,921	5,313
R-work/R-free (%)	18.9 / 21.3	17.8 / 19.7	17.7 / 19.9
**rms deviations (target in brackets)**			
Bond lengths (Å)	0.011 (0.013)	0.013 (0.013)	0.012 (0.013)
Bond Angles (°)	1.472 (1.648)	1.446 (1.648)	1.443 (1.648)
[1]Coordinate error (Å)	0.105	0.077	0.088
Mean B value ($Å^2$)	32.724	31.368	29.318
**Ramachandran Statistics (PDB Validation)**			
Favoured/allowed/Outliers	812 / 15 / 1	814/ 14 / 0	811 / 17 / 0
%	98.1 / 1.8 / 0.1	98.3 / 1.7 / 0	98 / 2 / 0

Figures in brackets refer to outer resolution shell, where applicable. [1] Coordinate Estimated Standard Uncertainty in (Å), calculated based on maximum likelihood statistics.

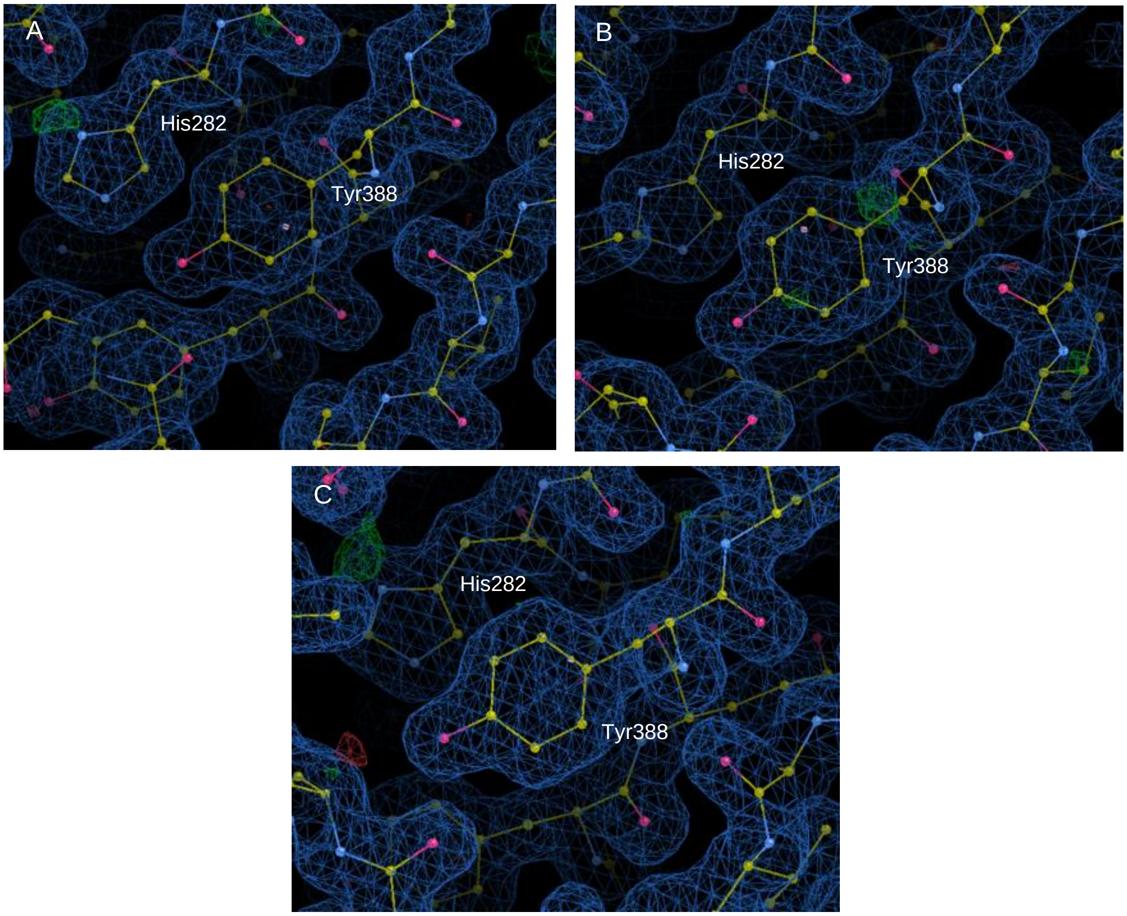

Figure S2. Electron density map and model of the XFEL structure of Tpp49Aa1. A slice through the centre of the electron density of a monomer of Tpp49Aa1 is shown. Protein backbone is coloured yellow, nitrogen atoms are coloured blue, and oxygen atoms are coloured pink. **(A)** Tpp49Aa1, pH 7 **(B)** Tpp49Aa1, pH 3 **(C)** Tpp49Aa1, pH 11

Table S3. Structural similarity of Tpp49Aa1 with other β-sheet toxins. Z-scores larger than 2 are considered significant.

PDB ID	Name (formally)	Z-score	RMSD (Å)
5FOY_B	Tpp2Aa2 (BinBA2)	41.4	2.1
3WA1_A	Tpp2Aa3 (BinBA3)	38.3	2.3
5FOY_A	Tpp1Aa2 (BinAA2)	37.9	2.5
8BAD_A	Tpp80Aa1 (Cry80Aa1)	33.4	4.2
4JP0_A	Tpp35Ab2 (Cry35Ab2)	29.3	3.5

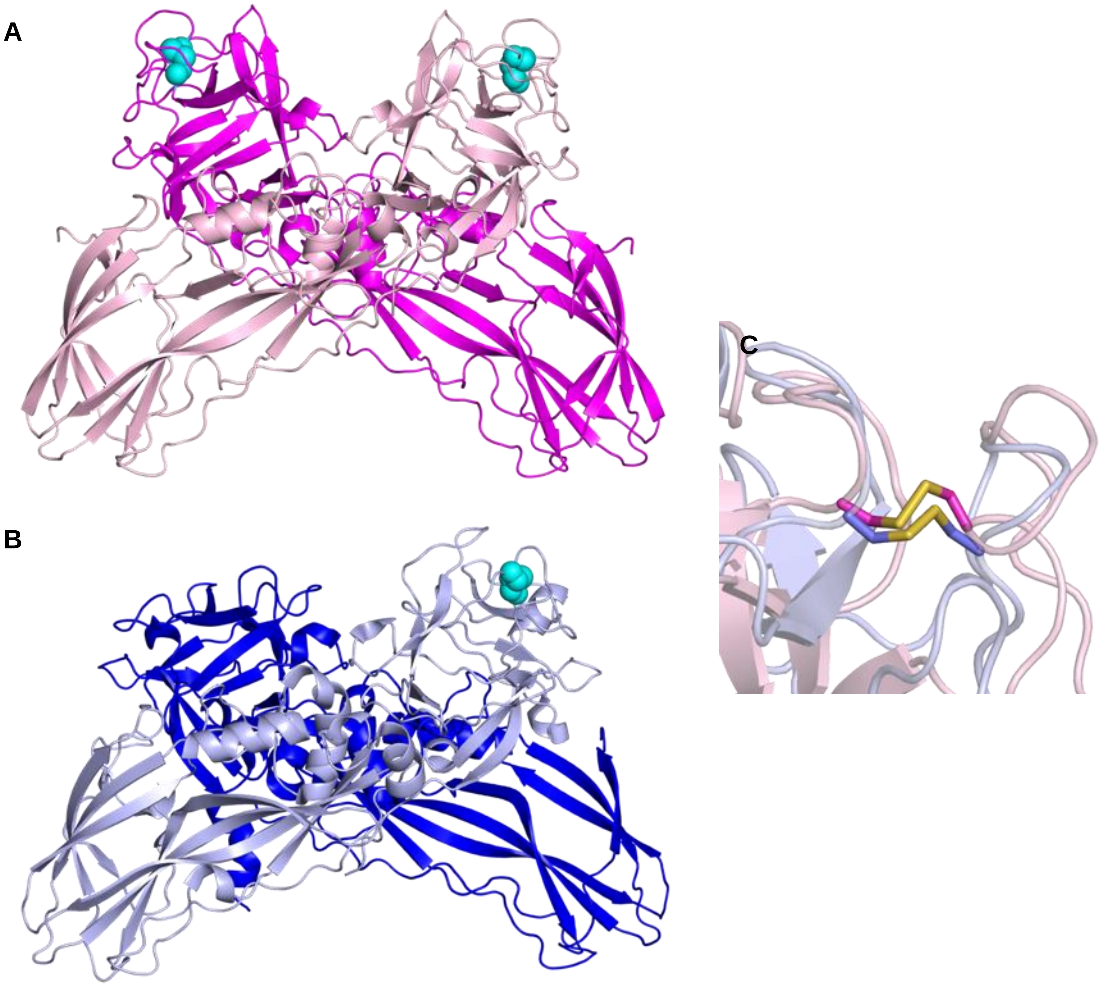

Figure S3. Tpp49Aa1 forms a homodimer similar to the Tpp1Aa2/Tpp2Aa2 heterodimer. (A) Tpp49Aa1 homodimer (magenta and light pink). **(B)** Tpp1Aa2 (blue)/Tpp2Aa2 (light blue) heterodimer. Equivalent disulphide bonds in Tpp49Aa1 and Tpp2Aa2 monomers are shown as spheres (cyan). In the Tpp49Aa1 homodimer, the interface between the two monomers involves 41 residues from monomer A and 42 residues from monomer B, making 16 hydrogen bonds. Interface analysis by PISA estimates the Tpp49Aa1 interface area at 1329.1 $Å^2$ and the binding energy at -11.1 kcal mol^{-1}. In the Tpp1Aa2/Tpp2Aa2 heterodimer, the interface between the Tpp1Aa2 and Tpp2Aa2 monomers involves 49 residues from Tpp1Aa2 and 63 residues from Tpp2Aa2, making 19 hydrogen bonds and 2 salt bridges. Consistent with this, interface analysis by PISA estimates the interface area at 1833.1 $Å^2$ and the binding energy at -22.5 kcal mol^{-1}, indicating a more stable complex for Tpp1Aa2/Tpp2Aa2 heterodimers than for Tpp49Aa1 homodimers. The RMSD of the aligned atoms between the Tpp49Aa1 homodimer and Tpp1Aa2/Tpp2Aa2 heterodimer was estimated by PyMOL at 9.023 Å. **(C)** Alignment of Tpp49Aa1 (light pink) and Tpp2Aa2 (light blue). The equivalent Tpp49Aa1 Cys91-Cys183, and Tpp2Aa2, Cys67-Cys161 disulphide bonds are shown as sticks.

A

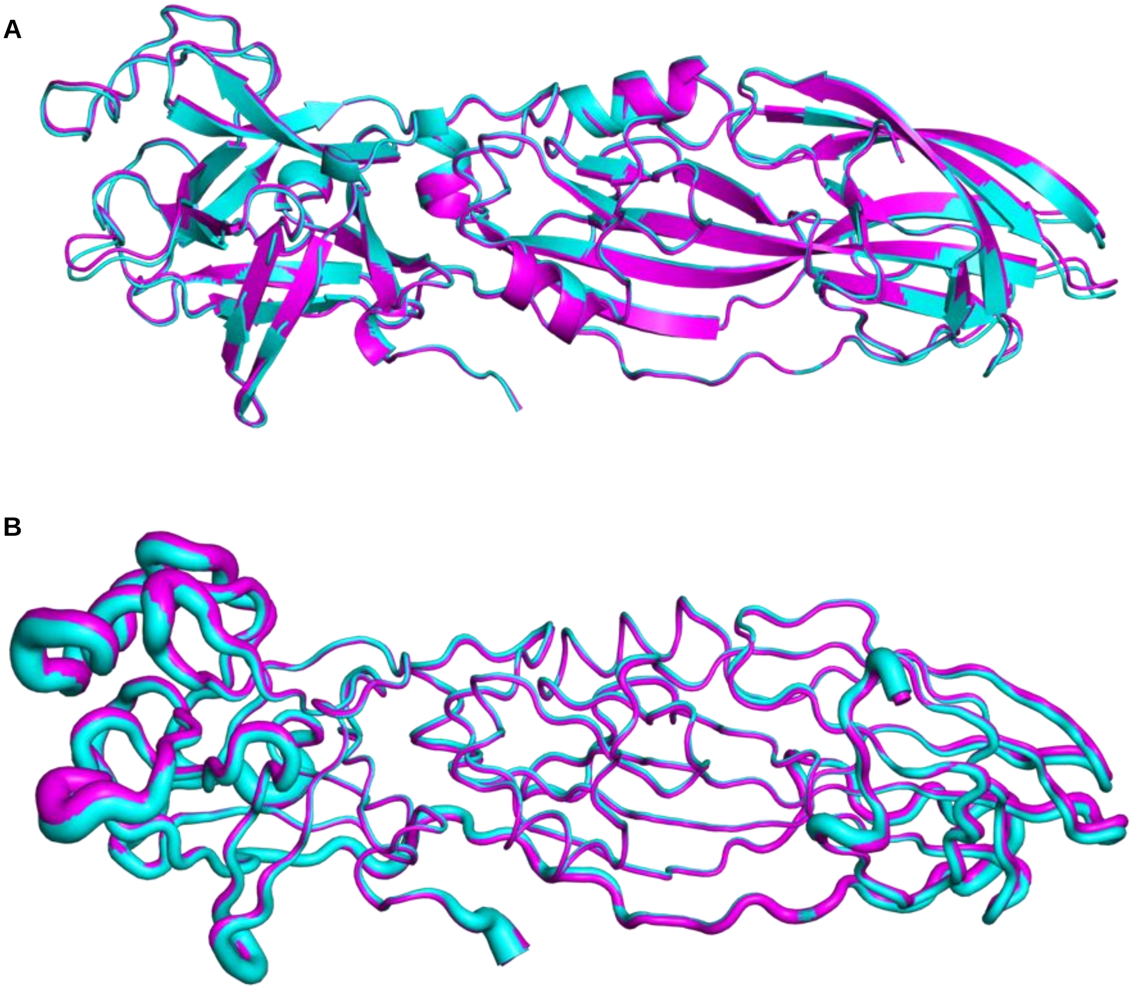

B

Figure S4. Superposition of the Tpp49Aa1 monomers. Showing the two copies to be almost identical, with an all-atom RMSD of 0.681 Å. **(A)** Superposition of Tpp49Aa1 monomers, represented as a cartoon. **(B)** Superposition of Tpp49Aa1 monomers, represented by B-factor putty. The B-factor, or thermal parameter, describes the magnitude of displacement of the atoms from their central positions.

Table S4. Structural properties of the molecular interfaces in the Tpp49Aa1 crystal at pH 7

Interface	Monomer 1			Monomer 2			Interface area (Å^2)	Δ^iG** (kcal mol^{-1})	H-bonds	Salt bridges	Binding energy (kcal mol^{-1})
	Chain	Symmetry	Nres	Chain	Symmetry	Nres					
1*	B	x,y,z	42	A	x,y,z	41	1329.1	-4.0	16	0	-11.1
2	A	x,y,z	13	B	x-1/2,-y-1/2,-z	13	470.2	-0.6	10	2	-5.8
3	B	x,y,z	12	A	-x,y-1/2,-z-1/2	13	445.1	-0.3	7	1	-3.8
4	B	x,y,z	12	A	-x+1/2,-y,z-1/2	10	383.4	-8.0	0	0	-8.0
5	B	x,y,z	20	B	x-1/2,-y-1/2,-z	15	361.3	-0.1	5	0	-2.3
6	A	x,y,z	12	A	-x,y-1/2,-z-1/2	8	147.9	0.9	1	0	0.5
7	A	x,y,z	6	B	x-1,y,z	6	169.1	-1.3	0	1	-1.7
8	A	x,y,z	3	B	-x-1/2,-y-1,z-1/2	3	91.6	0.5	1	3	-1.0
9	B	x,y,z	1	A	x,y-1,z	2	12.4	0.3	0	0	0.3
Total***	–	–	–	–	–	–	2081	-8.6	24	7	-21.8

Table S5. Structural properties of the molecular interfaces in the Tpp49Aa1 crystal at pH 3

Interface	Monomer 1			Monomer 2			Interface area (Å^2)	Δ^iG** (kcal mol^{-1})	H-bonds	Salt bridges	Binding energy (kcal mol^{-1})
	Chain	Symmetry	Nres	Chain	Symmetry	Nres					
1*	B	x,y,z	40	A	x,y,z	40	1333.5	-5.0	16	0	-12.1
2	A	x,y,z	14	B	x-1/2,-y-1/2,-z	14	466.5	-1.1	6	0	-3.8
3	B	x,y,z	12	A	-x,y-1/2,-z-1/2	13	445.0	-0.4	9	0	-4.4
4	B	x,y,z	10	A	-x+1/2,-y,z-1/2	11	354.0	-7.7	0	0	-7.7
5	B	x,y,z	18	B	x-1/2,-y-1/2,-z	11	305.2	1.9	3	0	0.5
6	A	x,y,z	11	A	-x,y-1/2,-z-1/2	6	145.4	1.1	1	0	0.6
7	A	x,y,z	6	B	x-1,y,z	5	113.7	-1.0	0	1	-1.3
8	A	x,y,z	3	B	-x-1/2,-y-1,z-1/2	3	89.2	1.1	1	4	-0.9
9	B	x,y,z	1	A	x,y-1,z	2	7.1	0.2	0	0	0.2
Total***	–	–	–	–	–	–	1926.1	-5.9	20	5	-16.8

Table S6. Structural properties of the molecular interfaces in the Tpp49Aa1 crystal at pH 11

Interface	Monomer 1			Monomer 2			Interface area (Å^2)	Δ^{i}G** (kcal mol^{-1})	H-bonds	Salt bridges	Binding energy (kcal mol^{-1})
	Chain	Symmetry	Nres	Chain	Symmetry	Nres					
1*	B	x,y,z	38	A	x,y,z	41	1312.8	-5.2	15	0	-11.9
2	A	x,y,z	13	B	x-1/2,-y-1/2,-z	13	477.8	0.2	9	1	-4.2
3	B	x,y,z	13	A	-x,y-1/2,-z-1/2	13	458.3	-1.0	7	1	-4.5
4	B	x,y,z	12	A	-x+1/2,-y,z-1/2	11	396.4	-7.0	0	0	-7.0
5	B	x,y,z	19	B	x-1/2,-y-1/2,-z	15	355.3	-0.5	4	0	-2.3
6	A	x,y,z	10	A	-x,y-1/2,-z-1/2	7	131.5	0.9	1	0	0.5
7	A	x,y,z	6	B	x-1,y,z	6	180.1	-1.4	0	1	-1.7
8	A	x,y,z	3	B	-x-1/2,-y-1,z-1/2	3	89.6	0.5	1	3	-1.1
9	B	x,y,z	2	A	x,y-1,z	3	12.8	0.3	0	0	0.3
Total***	–	–	–	–	–	–	2101.8	-8	22	6	-20

* Interface 1 refers to the Tpp49Aa1 dimer interface
** Δ^{i}G = solvation free energy gain
*** Total excludes dimer interface

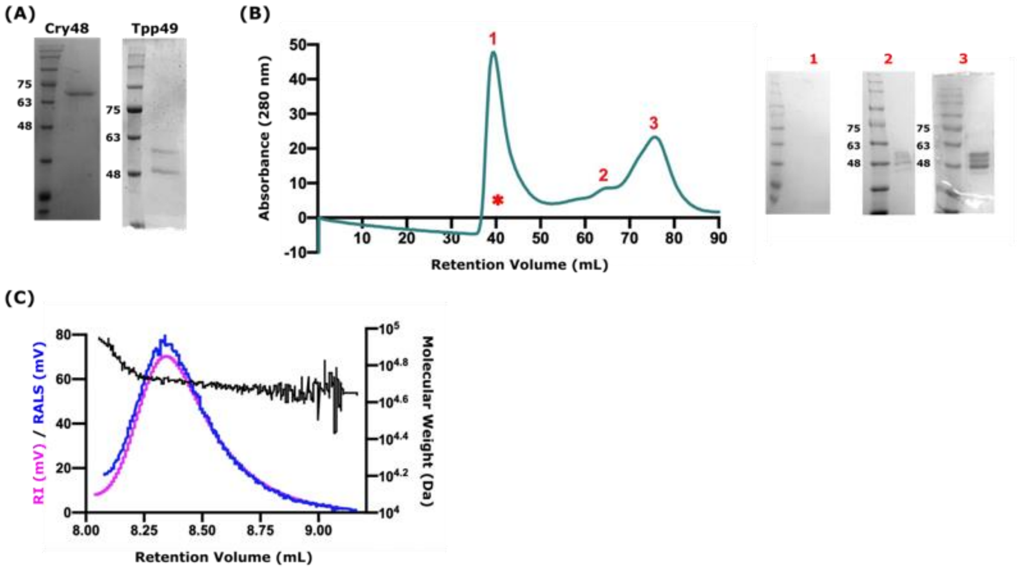

Figure S5. Tpp49Aa1 is predominantly monomeric in solution. A combination of size exclusion chromatography (SEC) and static light scattering (RALS) was used to determine if Tpp49Aa1 is dimeric or monomeric in solution. (**A)** Crystalline protein solubilised in Na_2CO_3 overnight showing Cry48Aa1 at ~ 70 kDa and Tpp49Aa1 as two bands at ~ 49 and ~55 kDa. (**B**) SEC shows three absorbance peaks (UV 280 nm) representing 766, 121, and 51 kDa, respectively. SDS-page resolution of the fractions taken from those peaks shows no product at 1 (*column void volume, presumably non-protein contaminants), and bands of approximately the expected sizes in 2 and 3. **(C)** Protein-containing peaks (2 & 3) were concentrated and used to decipher molecular weight via Right Angle Light Scattering (RALS, blue) and Refractive Index (RI, pink) measurements. From these data the molecular weight (Da, black) for the protein was calculated to be 52.1 kDa when calibrated to BSA (1mg/ mL) using OmniSEC software.

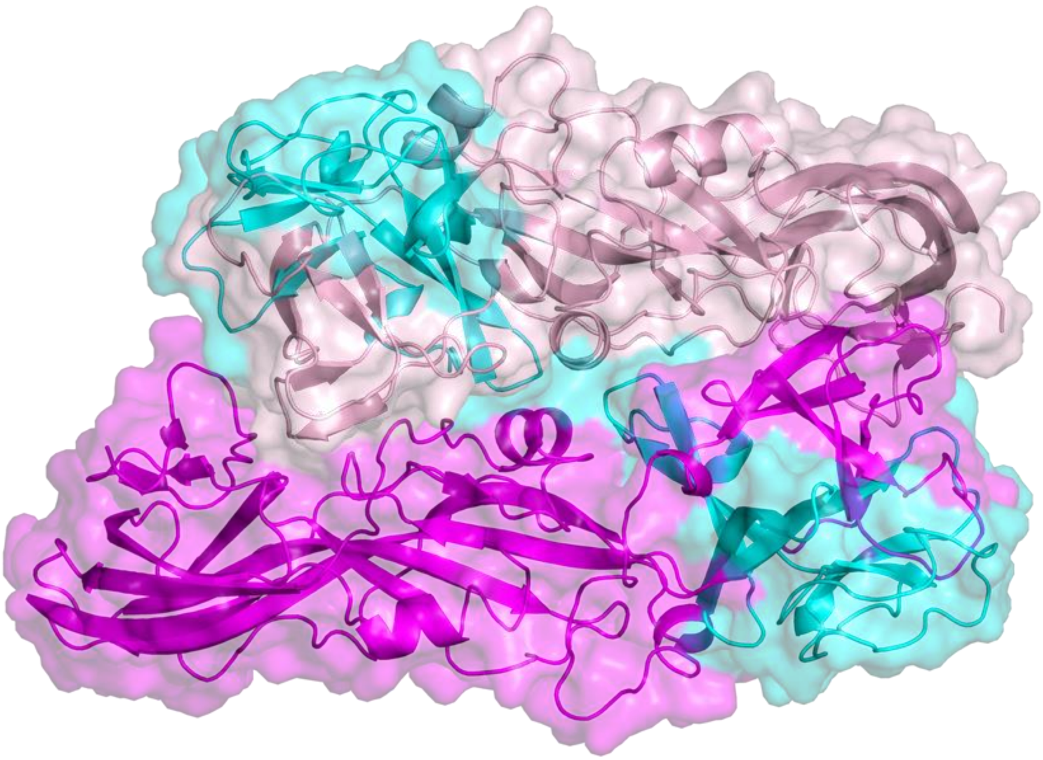

Figure S6. Tpp49Aa1 dimer highlighting the regions proposed to interact with Cry48Aa1. Tpp49Aa1 regions proposed to interact with Cry48Aa1 (cyan) are partially buried within the dimer interface. Tpp49Aa1 monomers shown in magenta and pink.

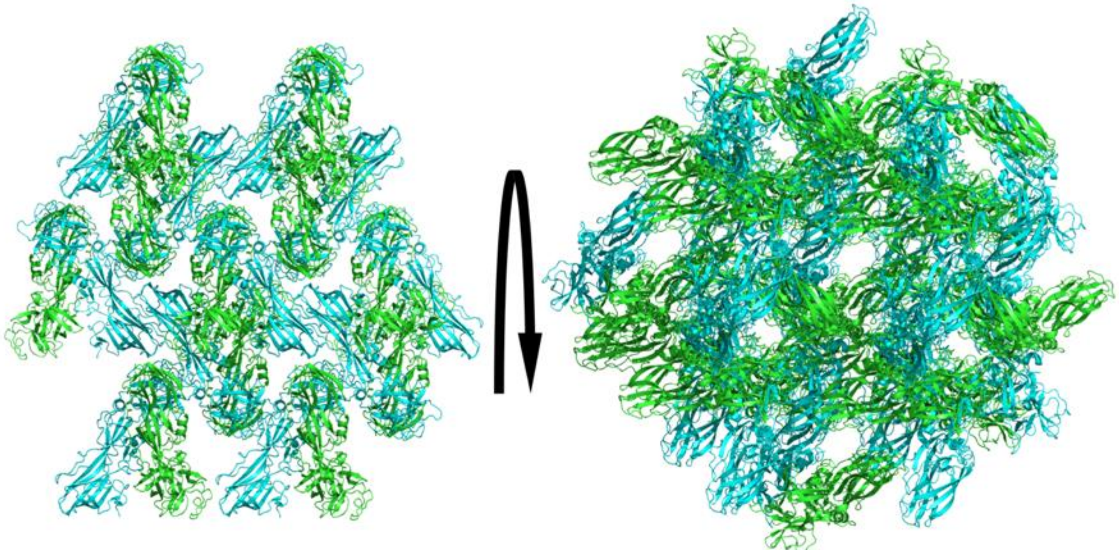

Figure S7. Solvent channels in Tpp49Aa1 crystals

Table S7. Loss/formation of interactions perturbed by an increase in pH from 7 to 11

**Interface**	Monomer 1 - symmetry	Monomer 2 - symmetry	Interactions
1*	x,y,z	x,y,z	Loss of:<br>- B:Asn51(ND2) – A:Asp347(O)<br>- B:Asn51(ND2) – A:Asn350(OD1)<br>- B:Gln162(OE1) – A:Asn357(ND2)<br>- B:Asn347(O) – A:Asn51(ND2)<br>- B:Asn350(OD1) – A:Asn51(ND2)<br>- B:Thr351(N) – A:Asn51(OD1)<br><br>Formation of:<br>- B:Asn51(ND2) – A:Thr351(O)<br>- B:Asp120(O) – A:Gln343(NE2)<br>- B:Gln343(NE2) – A:Asp120(O)<br>- B:Thr351(O) – A:Asn51(ND2)<br>- B:Tyr435(OH) – A:Asn165(O)
2	x,y,z	x-1/2,-y-1/2,-z	Loss of:<br>- A:Asp429(OD1) – B:Arg267(NH1) **<br>- A:Tyr463(O) – B:Thr400(OG1)
3	x,y,z	-x,y-1/2,-z-1/2	Loss of:<br>- B:Tyr463(O) – A:Thr400(OG1)<br><br>Formation of:<br>- B:Asp432(O) – A:Gln274(NE2)
4	x,y,z	-x+1/2,-y,z-1/2	
5	x,y,z	x-1/2,-y-1/2,-z	Loss of:<br>- B:Asp198(OD1) – B:Gln220(NE2)
6	x,y,z	-x,y-1/2,-z-1/2	
7	x,y,z	x-1,y,z	Loss of:<br>- A:His97(ND1) – B:Glu237(OE1)
8	x,y,z	-x-1/2,-y-1,z-1/2	Loss of:<br>- A:Asp88(N) – B:Asp88(OD1)
9	x,y,z	x,y-1,z	

* Interface 1 refers to the Tpp49Aa1 dimer interface
** Salt bridges

Table S8. Loss/formation of interactions perturbed by a decrease in pH from 7 to 3

Interface	Monomer 1 - symmetry	Monomer 2 - symmetry	Interactions
1*	x,y,z	x,y,z	Loss of:<br>- B:Gln162(OE1) – A:Asn357(ND2)<br><br>Formation of:<br>- B:Asn51(OD1) – A:Thr351(N)
2	x,y,z	x-1/2,-y-1/2,-z	Loss of:<br>- A:Asp429(OD1) – B:Arg267(NH1) **<br>- A:Asp429(OD2) – B:Arg267(NH1) **<br>- A:Ser434(N) – B:Gln274(OE1)<br>- A:Asp429(OD2) – B:Arg267(NH1)<br>- A:Asp429(O) – B:Arg267(NH1)<br>- A:Asp432(O) – B:Gln274(NE2)<br>- A:Tyr463(O) – B:Thr400(OG1)
3	x,y,z	-x,y-1/2,-z-1/2	Loss of:<br>- B:Asp429(OD2) – A:Arg267(NH2) **<br>- B:Asp429(OD2) – A:Arg267(NH2)<br><br>Formation of:<br>- B:Ser434(N) – A:Asp394(OD2)<br>- B:Asp432(O) – A:Gln274(NE2)
4	x,y,z	-x+1/2,-y,z-1/2	
5	x,y,z	x-1/2,-y-1/2,-z	Loss of:<br>- B:Arg196(NH2) – B:Gln220(OE1)<br>- B:Asn204(ND2) – B:Gln220(O)<br>- B:Asn206(ND2) – B:Asp394(O)<br><br>Formation of:<br>- B:Asn204(ND2) – B:Gln220(OE1)
6	x,y,z	-x,y-1/2,-z-1/2	
7	x,y,z	x-1,y,z	Loss of:<br>- A:His97(ND1) – B:Glu237(OE1) **<br>- A:His96(ND1) – B:Glu237(OE1)<br><br>Formation of:<br>- A:His97(NE2) – B:Glu237(OE2) **
8	x,y,z	-x-1/2,-y-1,z-1/2	Loss of:<br>- A:Asp88(N) – B:Asp88(OD1)<br><br>Formation of:<br>- A:Asp88(OD2) – B:Arg92(NE) **<br>- A:Asp88(N) – B:Asp88(OD2)
9	x,y,z	x,y-1,z	

* Interface 1 refers to the Tpp49Aa1 dimer interface
** Salt bridges

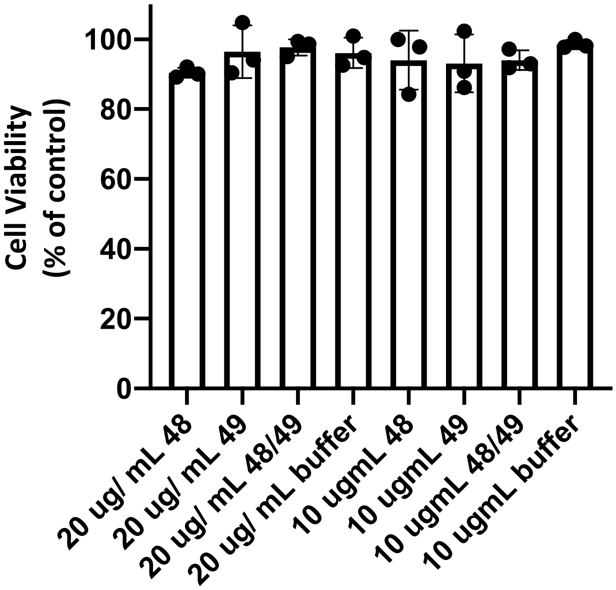

Figure S8. SF9 cells show no reduction in viability after 48-hour exposure to Tpp49Aa1/Cry48Aa1. Resazurin was used to quantify the effect of these proteins on cell viability. SF9 cells were treated with a range of concentrations of either Cry48Aa1 (“48”), Tpp49Aa1 (“49”), or equimolar amounts in combination (48/49). Resazurin was added to the cells 48 h post toxin exposure. No significant difference was observed. All data are presented as percentage of control conditions (buffer only) with the mean ± SD and all statistical analysis was performed using a one-way ANOVA.

Supplementary methods 1 - Size Exclusion Chromatography

For size exclusion chromatography (SEC), Cry48Aa1 and Tpp49Aa1 protein crystals were solubilised in 50 mM Na_2CO_3 pH 10.5 + 0.05% β-mercaptoethanol overnight at room temperature, with agitation. Insoluble material was removed by centrifugation, and the solubilised proteins buffer exchanged into 20 mM TrisHCl pH 8.5. SEC was performed using a calibrated Hiload™ 16/60 Superdex™ S200 pg column. For column calibration, BioRad standard proteins (of molecular weights 670, 158, 44, 17, and 1.35 kDa) were run on the column at a flow rate of 0.5 mL/min with 50 mM TrisHCl buffer pH 8. All protein samples were run on the column at a flow rate of 1 mL/min with 20 mM TrisHCl pH 8.5. Elution fractions were collected and analysed using SDS-PAGE.

Supplementary methods 2 - Static Light Scattering

To investigate whether Tpp49Aa1 was present as a monomer in solution we used static light scattering (RALS) and refractive index (RI) measurements. The Zetasizer MicroV system (Malvern Instruments Ltd., Malvern, UK) was used to quantify the size distribution of Tpp49Aa1 particles based upon time-dependent fluctuations in the scattered light intensity (at 90° scattering angle), due to the Brownian motion of the protein in solution. Concentration was determined by a refractive index (RI) detector (VE 3580, Viscotek Corp). Protein samples (100 µL at 1 mg/ mL) were prepared and solubilised as previously described, with protein-containing fractions pooled and concentrated from the first round of SEC (supplementary methods 1). The sample was again separated via SEC using a Superdex 75 Increase 10/300 GL with a flow rate of 0.8 mL/min, coupled to the Zetasizer and the refractive index (RI) detector. Eluted samples were measured every ~3 s at 30°C. Data were collected and analysed in OmniSEC software (Ver 5.12) and calibrated to BSA (1 mg/ mL).
